# An Elementary Treatise on Midwifery, or Principles of Toxology and Embryology

**Published:** 1845-12

**Authors:** 


*An Elementary Treatise on Midwifery, or Principles of Toxology and Embryology, by* Alf. A. L. M. Velpeau, M. D., translated from the French by Charles D. Meigs, M. D. &c. &c. Third American Edition, with notes and additions, by William Harris, M. D. &c. Philadelphia: Lindsay & Blakiston, 1845, octavo, pp. 600.

To commend Velpeau's Midwifery, were indeed to gild refined gold. The reputation of the author is fully established, and his work on Midwifery is admitted to be inferior to no elementary treatise or text book ever offered to the public. The notes of Dr. Meigs and Dr. Harris add to the completeness of the work. The essay on puerperal fever by Dr. Harris, is particularly worthy of notice. The subject itself, is full of interest, and the plain account of the disease, with the practical observations condensed in this short essay, add no little to the superior value of this edition to those which have preceded it.

M. Velpeau is not only a teacher of voice and pen; his example should stimulate every student and physician to exert his abilities to the utmost for the acquisition and diffusion of knowledge. He was an obscure man, with no friend but his own indomitable energy, no capital but his natural talents, yet he has, as Dr. Harris expresses it, "worked himself to enviable professsional eminence." He early adopted the line of conduct indicated by his noble sentiment, "the sciences compose a republic in which every man is at liberty to make researches, to examine and think for himself, as well as to say what he thinks." Truth is the avowed object of all who cultivate them; it may be reached by a hundred different routes; and I never could understand how any reasonable man could be offended because his ideas fail to be received as laws for other men.

M. Velpeau has the independence to think for himself, which in this age of servile obsequiousness, is strange. He has also, the ability to think for himself, which is stranger; and he has the industry to prosecute his own investigations and write out his own opinions, which is the strangest of all. In six years he made dissections of one hundred and forty fœtuses within the third month of gestation only. This one item of anatomical labor, will give some idea of the amount of work which M. Velpeau sustains and which sustains M. Velpeau. He is fifty years of age and has written for the press more than 25,000 pages. He has published 72 medical works, besides 140 other articles and memoirs, which are ascribed to him. Nevertheless, this extraordinary man finds time to lecture and to practice.

Honor to M. Velpeau: let the student who enters into his labors imitate his example.

---

